# A prospective study of differences in duodenum compared to remaining small bowel motion between radiation treatments: Implications for radiation dose escalation in carcinoma of the pancreas

**DOI:** 10.1186/1748-717X-1-33

**Published:** 2006-09-04

**Authors:** Anurag K Singh, Ryan M Tierney, Daniel A Low, Parag J Parikh, Robert J Myerson, Joseph O Deasy, Catherine S Wu, Gisele C Pereira, Sasha H Wahab, Sasa Mutic MS, Perry W Grigsby, Andrew J Hope

**Affiliations:** 1 Radiation Oncology Branch, National Cancer Institute, Bethesda, MD, 20892, USA; 2 Department of Radiation Oncology, Washington University Medical School, Saint Louis 63108, MO, USA

## Abstract

**Purpose:**

As a foundation for a dose escalation trial, we sought to characterize duodenal and non-duodenal small bowel organ motion between fractions of pancreatic radiation therapy.

**Patients and methods:**

Nine patients (4 women, 5 men) undergoing radiation therapy were enrolled in this prospective study. The patients had up to four weekly CT scans performed during their course of radiation therapy. Pancreas, duodenum and non-duodenal small bowel were then contoured for each CT scan. On the initial scan, a four-field plan was generated to fully cover the pancreas. This plan was registered to each subsequent CT scan. Dose-volume histogram (DVH) analyses were performed for the duodenum, non-duodenal small bowel, large bowel, and pancreas.

**Results:**

With significant individual variation, the volume of duodenum receiving at least 80% of the prescribed dose was consistently greater than the remaining small bowel. In the patient with the largest inter-fraction variation, the fractional volume of non-duodenal small bowel irradiated to at least the 80% isodose line ranged from 1% to 20%. In the patient with the largest inter-fraction variation, the fractional volume of duodenum irradiated to at least the 80% isodose line ranged from 30% to 100%.

**Conclusion:**

The volume of small bowel irradiated during four-field pancreatic radiation therapy changes substantially between fractions. This suggests dose escalation may be possible. However, dose limits to the duodenum should be stricter than for other segments of small bowel.

## Background

The annual incidence of pancreatic cancer is approximately 30,000 [1]. For these patients, 5 year survival is less than 5%[1]. Despite advances in chemotherapeutic agents and radiation therapy techniques, there are virtually no long term survivors among patients unable to undergo surgical resection[1].

In principle, increasing the amount of radiation delivered to the pancreas may improve these dismal results. Unfortunately, the pancreas moves substantially with respiration[2], and the surrounding organs, notably stomach and small bowel, undergo significant volume changes during radiation therapy [3].

Such pancreatic motion and change in surrounding normal small bowel makes dose escalation problematic. A variety of strategies are being explored to quantify and address issues relating to pancreatic motion, including gating of radiation with respiration[4] and implantation of markers to directly track organ movement[5]. However, little work has been done to address the issue of quantifying and limiting dose to the small bowel.

Much of the small bowel experiences complex motion. The duodenum, however, is relatively fixed.

This study sought to quantify volume changes of the duodenum and non-duodenal small bowel within a standardized pancreatic treatment volume.

## Patients and methods

Nine patients undergoing radiation therapy – four with cervical cancer and five with prostate cancer – were prospectively enrolled onto an intramural protocol. Each underwent CT simulation in the supine position in an alpha cradle fixed to the treatment table in order to minimize daily setup variation. An external radioopaque fiducial marker was embedded into the cradle in the midline superior to each patient's iliac crest around the L3 vertebral level. The patients then had a weekly CT scan performed for up to four weeks during their course of radiation therapy to determine inter-fraction motion. The patients were breathing freely and were given no instructions regarding time from their last meal to the next scheduled treatment time. Small bowel contrast was given for all patients except for two who could not tolerate the contrast administration. No spasmolytics were used.

The whole pancreas was contoured, and this contour was designated gross tumor volume (GTV). Small bowel was contoured as duodenum and non-duodenal small bowel. Specifically, the duodenum was contoured from the pylorus to its ascending (fourth) portion, lateral to the head of the pancreas. All remaining small bowel to the level of the ascending colon was designated "small bowel, excluding duodenum." Bowel was contoured closely along the wall of each loop on each axial slice for each scan generated. The lumen was included in all bowel contours. All contouring was manual.

A four-field plan was generated based on the initial CT scan. The four equally-weighted fields consisted of two AP/PA fields, measuring 15 × 10 cm, and two lateral fields, each measuring 10 × 10 cm. The treatment plans were designed to provide 100% coverage to the GTV based on the initial CT scan. The initial treatment plan was then run on each subsequent CT scan for each patient. The prescription dose was 50.4 Gy in 1.8 Gy daily fractions.

All treatment plans were transferred using the RTOG QA protocol to an custom treatment planning research software platform, the Computational Environment for Radiotherapy Research (CERR)[6]. CERR includes: (1) an import program which converts the widely available AAPM/RTOG treatment planning format into a MATLAB cell-array data object, facilitating manipulation; (2) viewers which display axial, coronal, and sagittal computed tomography images, structure contours, digital films, and isodose lines or dose colorwash, (3) a suite of contouring tools to edit and/or create anatomical structures, and (4) dose-volume and dose-surface histogram calculation and display tools.

The plan and its resultant dose distributions were registered to each subsequent CT scan by keeping constant the relative position between the beam isocenter and the external fiducial marker embedded in the alpha cradle. DVH and organ motion analyses were performed on the GTV, the duodenum, non-duodenal small bowel, and large bowel. Total volume of these organs was noted for each CT scan (see Table 1.).

**Table 1:** Listing of mean, minimum, and maximum CT volume, in cubic centimeters, of duodenum and small bowel (excluding duodenum) for all study patients.

**Patient #**	**Duodenum Volume Mean (Range)**	**Non-duodenal Small Bowel Volume Mean (Range)**
**1**	73 (58–110)	3182 (2062–3657)
**2**	111 (88–135)	1195 (1128–1261)
**3**	58 (55–62)	1435 (1337–1504)
**4**	44 (30–58)	954 (915–993)
**5**	32 (27–34)	1042 (989–1079)
**6**	31 (16–45)	1186 (1102–1335)
**7**	37 (31–44)	756 (673–867)
**8**	54 (21–120)	810 (721–1034)
**9**	8 (7–9)	655 (448–858)

## Results

**Figure 1:**
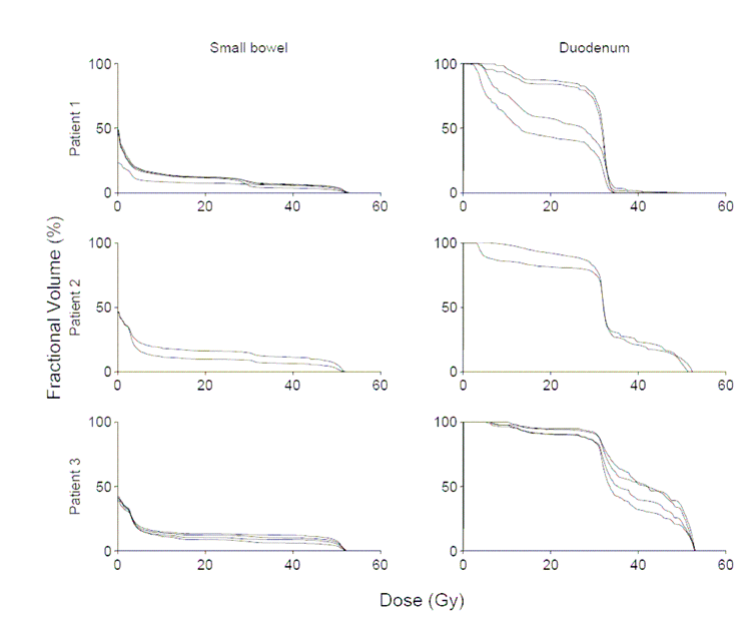
Small bowel (excluding duodenum), and duodenum dose-volume histograms for all available fractions in patients 1–3.

**Figure 2:**
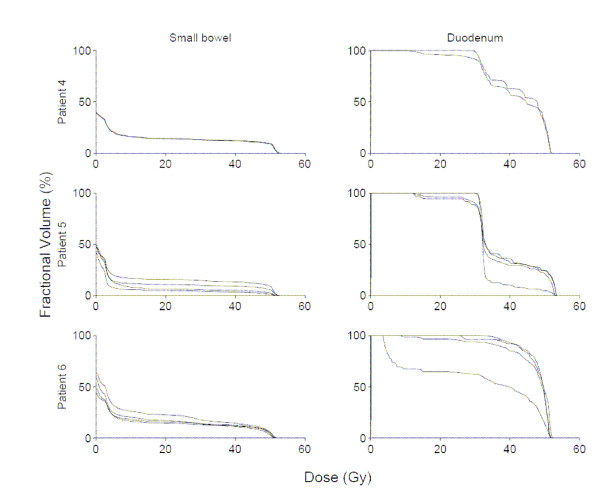
Small bowel (excluding duodenum), and duodenum dose-volume histograms for all available fractions in patients 4–6.

**Figure 3:**
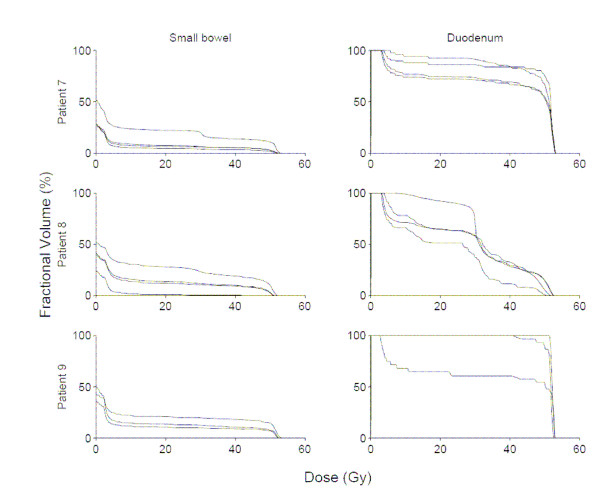
Small bowel (excluding duodenum), and duodenum dose-volume histograms for all available fractions in patients 7–9.

Among these 9 patients, 31 total CT scans were available for analysis. Figures 1, 2, 3 show DVHs from all patients. These data illustrate the effects of inter-fraction motion on coverage of duodenum and small bowel (excluding duodenum). The fractional volume of non-duodenal small bowel receiving at least 80% (40 Gy) of the prescribed dose to the pancreas varied significantly in individual patients. Specifically, in the patient with the largest inter-fraction variation, the fractional volume of small bowel irradiated to at least 40 Gy ranged from 1% to 20%. In the patient with the smallest inter-fraction variation, the fractional volume of small bowel irradiated to at least 40 Gy ranged from 11% to 12%. Both the absolute volume and inter-fraction variation of duodenum irradiated to at least 40 Gy differed significantly compared with the rest of the small bowel. In the patient with the largest inter-fraction variation, the fractional volume of duodenum irradiated to at least 40 Gy ranged from 30 to 100%. In the patient with the smallest inter-fraction variation, the fractional volume of duodenum irradiated to at least 40 Gy ranged from 60% to 100%.

## Discussion

Our data present two unique findings. First, within a standardized pancreatic treatment volume, small bowel motion is substantial and only small volumes receive 80% of the prescribed dose. Second, our data suggests that relative motion of the duodenum is quite distinct from the remainder of the small bowel. Percent volumes of the duodenum receiving 80% of the prescribed dose were far greater than percent volumes of small bowel receiving the same dose.

This relative immobility of the duodenum compared with the remaining small bowel is a consequence of known anatomy. The duodenum is fixed to the pancreas (by the pancreatic duct) and to the gall bladder (by the common bile duct). Additionally, near the level of the pancreas, the duodenum is fixed by the ligament of Treitz, a suspensory muscle arising from the stems of celiac and superior mesenteric arteries and inserting into the third and fourth portions of the duodenum[7]. These attachments between duodenum and surrounding structures more directly limit motion of the duodenum than the rest of the small bowel. Consequently, the motion of the duodenum is different from the rest of the small bowel. This difference in motion between radiation treatments produces differences in the volume of tissue irradiated. To track these differences, the relatively fixed duodenum should be contoured as a separate structure from the more freely-moving remaining small bowel

Our data further suggests that the complex motion of the small bowel may be exploited for therapeutic benefit. The relative lack of anatomic attachments of the jejunum and ileum allow for complex motion. The result of this motion may be that irradiation of different segments of this non-duodenal small bowel occurs during each fraction. If different non-duodenal segments are being irradiated daily, then no single segment may get the full prescription dose. Therefore, dose escalation to the pancreas (using strategies such as implanted fiducial markers and/or respiratory gating to allow small fields while still allowing reliable tumor targeting despite motion) may be possible without a concomitant increase in non-duodenal small bowel toxicity.

Individual variations in our results make it impossible to suggest dose volume limits for either duodenum or small bowel. In fact, the individual variation in the data suggests that general guidelines may lack utility. Thus, recommendations may have to be individualized based on interfraction motion.

A trial of dose escalation, without chemotherapy, using three dimensional conformal radiation therapy to 70–72 Gy was performed in 44 patients with locally advanced pancreatic adenocarcinoma. The stomach and duodenum was limited to 50 Gy; however, given the aforementioned proximity to the pancreas, 30% of the duodenum was allowed to exceed this limit. Forty-one patients received the intended total dose. Treatment was never stopped because of toxicity. Acute Grade 3 toxicity was seen in 9% of patients. Late Grade 3 and Grade 4 gastrointestinal toxicity was seen in 3 patients and 2 patients, respectively. Late (Grade 5) gastrointestinal bleeding was observed in 3 patients, 2 of whom had local tumor progression. Local disease progression was observed in 44% of patients. No true partial or complete responses were documented. The median survival from the time of diagnosis was 10 months from the start of radiotherapy. Distant metastases remained the major problem.[8]

The inability of 70 Gy to achieve local control may reflect an insufficient dose or an inability to irradiate the pancreas due to organ motion. Well characterized by several previously published reports, [2-4] our data (not shown) also suggests that pancreatic motion is significant and needs to be accounted for with any conformal technique.

We trust that existing and developing technology and methodology will allow reliable irradiation of the pancreas with small fields despite organ motion. Existing literature shows dismal outcomes with conventional therapy and the feasibility of dose escalation to 70 Gy. Therefore trials of radiation dose escalation beyond the conventional 50 Gy, possibly with concurrent chemotherapy, remain reasonable in inoperable pancreatic cancer. As issues relating to pancreatic motion are addressed, we hope future pancreatic dose escalation studies will track irradiated volume of duodenum separately from the remainder of the small bowel and report correlations with toxicity.

## Conclusion

The volume of irradiated small bowel excluding duodenum changes significantly between fractions of pancreatic radiation therapy. These relatively large volume changes suggest that dose escalation to the pancreas may be possible, as no single segment of non-duodenal small bowel is likely to receive the full prescription dose. However, the volume of irradiated duodenum is relatively more stable. Therefore, dose/volume constraints on the duodenum should be more stringent than for the remaining small bowel.

## Competing interests

The author(s) declare that they have no competing interests.

## Meeting presentation

A portion of this work was presented at the 90th Scientific Assembly and Annual Meeting of the Radiological Society of North America, Chicago, IL, Nov 28-Dec 3, 2004.
